# Therapeutic efficacy of matrix metalloproteinase-12 suppression on neurological recovery after ischemic stroke: Optimal treatment timing and duration

**DOI:** 10.3389/fnins.2022.1012812

**Published:** 2022-10-04

**Authors:** Siva Reddy Challa, Koteswara Rao Nalamolu, Casimir A. Fornal, Billy C. Wang, Ryan C. Martin, Elsa A. Olson, Ammar L. Ujjainwala, David M. Pinson, Jeffrey D. Klopfenstein, Krishna Kumar Veeravalli

**Affiliations:** ^1^Department of Cancer Biology and Pharmacology, University of Illinois College of Medicine at Peoria, Peoria, IL, United States; ^2^Department of Pharmacology, KVSR Siddhartha College of Pharmaceutical Sciences, Vijayawada, India; ^3^Department of Pediatrics, University of Illinois College of Medicine at Peoria, Peoria, IL, United States; ^4^Children’s Hospital of Illinois, OSF HealthCare Saint Francis Medical Center, Peoria, IL, United States; ^5^Department of Health Sciences Education and Pathology, University of Illinois College of Medicine at Peoria, Peoria, IL, United States; ^6^Department of Neurosurgery, University of Illinois College of Medicine at Peoria, Peoria, IL, United States; ^7^OSF HealthCare Saint Francis Medical Center, Illinois Neurological Institute, Peoria, IL, United States; ^8^Department of Neurology, University of Illinois College of Medicine at Peoria, Peoria, IL, United States

**Keywords:** ischemia, reperfusion, matrix metalloproteinase-12, sensory, motor, recovery

## Abstract

We recently showed that the post-ischemic induction of matrix metalloproteinase-12 (MMP-12) in the brain degrades tight junction proteins, increases MMP-9 and TNFα expression, and contributes to the blood-brain barrier (BBB) disruption, apoptosis, demyelination, and infarct volume development. The objectives of this study were to (1) determine the effect of MMP-12 suppression by shRNA-mediated gene silencing on neurological/functional recovery, (2) establish the optimal timing of MMP-12shRNA treatment that provides maximum therapeutic benefit, (3) compare the effectiveness of acute versus chronic MMP-12 suppression, and (4) evaluate potential sex-related differences in treatment outcomes. Young male and female Sprague-Dawley rats were subjected to transient middle cerebral artery occlusion and reperfusion. Cohorts of rats were administered either MMP-12shRNA or scrambled shRNA sequence (control) expressing plasmids (1 mg/kg; i.v.) formulated as nanoparticles. At designated time points after reperfusion, rats from various groups were subjected to a battery of neurological tests to assess their reflex, balance, sensory, and motor functions. Suppression of MMP-12 promoted the neurological recovery of stroke-induced male and female rats, although the effect was less apparent in females. Immediate treatment after reperfusion resulted in a better recovery of sensory and motor function than delayed treatments. Chronic MMP-12 suppression neither enhanced nor diminished the therapeutic effects of acute MMP-12 suppression, indicating that a single dose of plasmid may be sufficient. We conclude that suppressing MMP-12 after an ischemic stroke is a promising therapeutic strategy for promoting the recovery of neurological function.

## Introduction

Ischemic stroke remains the most prevalent form, accounting for about 87% of all strokes. The only two FDA-approved recanalization treatments for ischemic stroke are thrombolysis drug therapy with tissue-type plasminogen activator and endovascular thrombectomy. Following the recanalization treatments, blood flow is restored, ending ischemia. Nonetheless, both the residual brain damage and that resulting from recanalization (reperfusion injury) contribute to the development of severe and long-lasting secondary effects, including brain injury and neurologic functional deficits. Consequently, it is of utmost clinical importance to discover new treatments that prevent brain damage and promote the recovery of sensory and motor function. Despite years of intensive research, drug treatments are not available to alleviate progressive brain damage or enhance neurologic functional recovery after an acute ischemic stroke.

Recently, we demonstrated the upregulation of matrix metalloproteinase-12 (MMP-12) in the ischemic brain of young rodents during both the acute and chronic phases following an ischemic stroke (Chelluboina et al., 2015b; Nalamolu et al., 2018). We discovered that the upregulation of MMP-12 in the ischemic brain was approximately 8–200 times greater than any other MMP. MMP-12 expression increased gradually during the first week following transient focal cerebral ischemia and reperfusion, and remained elevated for 14 days, the longest post-reperfusion duration tested in our study (Chelluboina et al., 2015b). MMP-12 possesses autoproteolytic properties and can activate other MMPs (Chen, 2004). It can activate pro-MMP-2 and pro-MMP-3, which can then activate pro-MMP-1 and pro-MMP-9 (Matsumoto et al., 1998). MMP-2 and MMP-9 are the primary mediators of blood-brain barrier (BBB) disruption because they degrade various components of the microvascular basal lamina and BBB tight junction proteins (Rosenberg, 2002; Zhao et al., 2006; Yang et al., 2007; del Zoppo, 2009). In addition, there is evidence that MMP-9 plays a significant role in vasogenic brain edema and secondary brain damage (Montaner et al., 2001). MMP-12 activation has been reported to induce myelin basic protein degradation (Chandler et al., 1996). Demyelination is a major component of white matter injury. It significantly contributes to long-term sensorimotor and cognitive deficits. MMP-12 can induce the release of TNFα from macrophages, thereby initiating the inflammatory cascade (Churg et al., 2003). Animals lacking MMP-12 have defective TNFα release, and MMP-12 can act as a converting enzyme to convert pro-TNFα to active TNFα (Chandler et al., 1996; Churg et al., 2003). The intracellular levels of MMP-12 contribute to the secretion of IFN-α, a master cytokine capable of boosting the production of other pro-inflammatory cytokines, such as IL-1, IL-2, IL-6, TNFα, and IFN-γ, whereas the extracellular MMP-12 cleaves the IFN-α receptor 2–binding site of systemic IFN-α (Marchant et al., 2014). Progranulin (PGRN) is a growth factor and a source of various inflammatory mediators after undergoing proteolysis to granulins. MMP-12 has been identified as a proteolytic enzyme of PGRN, which is widely expressed in mammalian tissues (Bhandari et al., 1992; Daniel et al., 2000, 2003; Mackenzie et al., 2006; Matsubara et al., 2012). In conclusion, the plausible molecular interactions discussed above in relation to elevated MMP-12 levels in the brain may significantly contribute to the pathophysiology of ischemic stroke and poor stroke recovery.

Reducing MMP-12 expression in the brain via shRNA-mediated gene silencing mitigated ischemic brain damage, at least in part, by reducing the degradation of tight junction proteins, MMP-9 elevation, BBB disruption, apoptosis, myelin basic protein degradation, TNFα upregulation, and infarct volume (Chelluboina et al., 2015a,b). In light of these promising findings, we investigated whether suppressing MMP-12 in the brain after an ischemic stroke might have a beneficial effect on stroke recovery and therefore be a potential stroke treatment.

This study had the following objectives: (1) determine the effect of MMP-12 suppression on neurological and functional recovery in stroke-induced male and female animals; (2) determine the optimal timing of MMP-12shRNA treatment for maximum therapeutic benefit; (3) compare the therapeutic efficacy of acute versus chronic MMP-12 suppression; and (4) identify any treatment-related sex differences on functional outcomes.

## Materials and methods

### Design, construction, and synthesis of matrix metalloproteinase-12 shRNA plasmids

**FIGURE 1:**
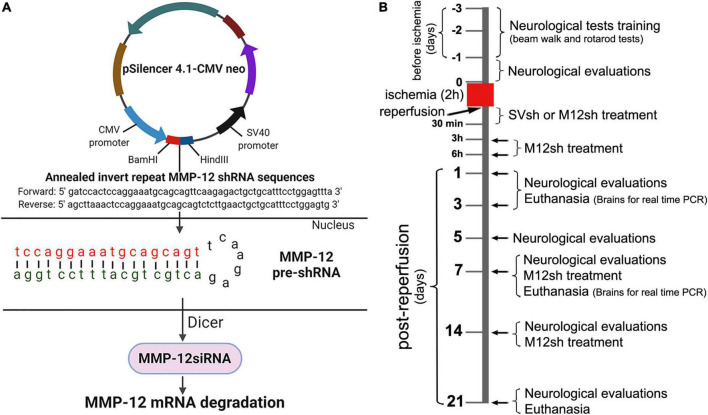
Schematic representation of the MMP-12 shRNA plasmid construct and mechanism of target gene silencing, experimental design, and timing of the main experimental procedures. **(A)** The construct consists of the annealed invert repeat MMP-12 shRNA sequences ligated between *Bam*HI and *Hin*dIII sites of the pSilencer 4.1-CMV neo expression vector. Following the entry of plasmids into the nucleus of a cell, the CMV promoter drives the formation of the MMP-12 pre-shRNA, a short hairpin molecule specific to MMP-12. The Dicer processes MMP-12 pre-shRNA molecules and the resulting MMP-12 siRNA molecules interact with the target MMP-12 mRNA. This interaction leads to the degradation of MMP-12 mRNA and suppression of MMP-12 gene expression. **(B)** Rats from both sexes were subjected to transient focal cerebral ischemia for 2 h, followed by reperfusion and various treatments. Rats from the appropriate cohorts were euthanized on various post-reperfusion days, and their brains were processed for real-time PCR analysis. Additional animals from the appropriate cohorts were assessed in various neurological and functional tests at baseline and at regular intervals (post-reperfusion days 1, 3, 5, 7, 14, and 21) after a 2-h focal cerebral ischemia.

We designed, constructed, and synthesized plasmids expressing MMP-12 shRNA (M12sh) specifically to silence the gene expression of MMP-12. We utilized pSilencer™ 4.1-CMV neo vector obtained from Ambion (Austin, TX, USA) to construct M12sh along with a scrambled sequence shRNA (SVsh or control shRNA), which served as a control. The target MMP-12 mRNA sequence (actccaggaaatgcagcagttc) was chosen and used to design the inverted repeat MMP-12 shRNA sequences (Figure 1A). The inverted repeat sequences synthesized for MMP-12 were laterally symmetrical, causing them to be self-complementary with a nine base pair mismatch in the loop region. The oligonucleotides/ultramers were annealed, and the annealed product was ligated to the vector at the *Bam*HI and *Hin*dIII sites in accordance with the manufacturer’s instructions. Scrambled sequence shRNAs were also prepared in a similar manner. The resultant vectors were transformed into chemically competent *E. coli* cells (JM109 competent cells) and cultured overnight. Plasmids expressing M12sh or SVsh were synthesized from the overnight bacterial culture by using QIAGEN plasmid mini kit (Qiagen, USA) in accordance with the manufacturer’s protocol. Positive clones confirmed by gene sequencing analysis at the University of Illinois at Urbana-Champaign were used in this study.

The Institutional Biosafety Committee (IBC) of the University of Illinois College of Medicine Peoria approved the synthesis and isolation of plasmids containing shRNAs (control shRNA and MMP-12 shRNA) inserted in pSilencer™ 4.1-CMV neo vector (Ambion, Austin, TX, USA) from bacterial cultures and their use for several *in vitro* and *in vivo* experiments in our laboratory. The development and production of plasmids and their handling by research personnel were in compliance with the IBC-approved protocol.

### Induction of cerebral ischemia and reperfusion in animals

A total of 226 healthy young Sprague-Dawley rats (2–3 months old) were used in this study. Animals were procured (Envigo Laboratories, Indianapolis, IN, USA) and housed in the Laboratory Animal Care Facility at the University of Illinois College of Medicine Peoria. The housing conditions included a 12 h light/dark cycle, controlled temperature and humidity, and free access to food and water. Animals were randomly assigned to the different experimental groups [Sham, Untreated, SVsh-treated (SVsh-IAR/SVsh-SD and SVsh-RD), M12sh-treated (M12sh-IAR/M12sh-SD, M12sh-3hAR, M12sh-6hAR, and M12sh-RD)] as described in Table 1. To induce transient focal cerebral ischemia, 9–12 weeks-old rats were subjected to a suture model right middle cerebral artery occlusion (MCAO) as previously described by our group (Chelluboina et al., 2017). Rats in the sham group underwent the same surgical procedure as MCAO rats, but no sutures were inserted. Removal of the monofilament suture 2 h after MCAO constituted reperfusion. Animals from various cohorts were subjected to several testing procedures and/or drug treatments, before being euthanized at different time points following ischemia and reperfusion (Figure 1B).

**TABLE 1:** Experimental groups, description, and the number of animals used for each experiment.

Group name	Group description	Number of animals			
		PCR	Neurological studies	Mortality and exclusion	Total
**Sham**	Rats subjected to surgical procedure as MCAO rats, but no sutures were inserted.	6♂	–	–	6♂
**Untreated**	Rats subjected to MCAO but received no treatment	18♂	–	7♂	25♂
**SVsh treated** SVsh-IAR/ SVsh-SD	Rats subjected to MCAO and received SVsh treatment immediately (within 30 min) after reperfusion.	–	18♂ + 13♀	12♂ + 7♀	30♂ + 20♀
SVsh-RD	Rats subjected to MCAO and received SVsh treatment immediately (within 30 min) after reperfusion and on post-reperfusion days 7 and 14.	–	8♂	4♂	12♂
**M12sh treated** M12sh-IAR/ M12sh-SD	Rats subjected to MCAO and received M12sh treatment immediately (within 30 min) after reperfusion.	18♂	18♂ + 12♀	15♂ + 5♀	51♂ + 17♀
M12sh-3hAR	Rats subjected to MCAO and received M12sh treatment 3h after reperfusion.	–	12♂	5♂	17♂
M12sh-6hAR	Rats subjected to MCAO and received M12sh treatment 6h after reperfusion.	–	12♂	6♂	18♂
M12sh-RD	Rats subjected to MCAO and received M12sh treatment immediately (within 30 min) after reperfusion and on post-reperfusion days 7 and 14.	–	10♂ + 10♀	4♂ + 6♀	14♂ + 16♀

♂, Male; ♀, female; MCAO, middle cerebral artery occlusion; PCR, polymerase chain reaction; AR, after reperfusion; SD, single dose; RD, repeated dose.

As stated in the *Guide for the Care and Use of Laboratory Animals* (Publication no. 86–23 revised, National Institutes of Health, U.S. Department of Health and Human Services), all animal experiments were planned and conducted in accordance with the scientific, humane, and ethical principles. The Institutional Animal Care and Use Committee (IACUC) of the University of Illinois College of Medicine Peoria approved all surgical procedures and pre- and post-operative animal care. All animal experiments were conducted in accordance with the IACUC-approved animal protocol.

### Plasmid synthesis, formulation preparation, and treatment

The positive clones of M12sh and SVsh were inoculated into the sterilized Luria-Bertani media and cultured overnight at 37°C using an orbital shaker. After 16 h, the culture was centrifuged at 6000 × *g* for 15 min at 4°C to collect the pellet. The plasmid from the pellet was extracted using QIAGEN plasmid maxi kit (Qiagen, USA) in accordance with the manufacturer’s protocol. The obtained plasmids were stored at −20°C until use in nanoparticle formulation preparation. The M12sh or SVsh plasmids were formulated into nanoparticles, which are small enough to diffuse into tissues and enter cells by endocytosis. The nanoparticle formulation was prepared using the *in vivo*-jetPEI reagent (Polyplus transfection, Illkirch, France) in accordance with the manufacturer’s instructions. Nanoparticle formulations of M12sh or SVsh plasmids (1 mg/kg) were administered intravenously via the tail vein to rats in the respective groups at designated time points, including immediately (within 30 min), 3, and 6 h after reperfusion, as well as on post-reperfusion days 7 and 14 as described in Table 1.

### Real-time PCR analysis

Total RNA was extracted from the whole contralateral and ipsilateral brains of male rats from appropriate cohorts using TRIzol reagent (Invitrogen, Carlsbad, CA, USA). One microgram of total RNA from each sample was reverse transcribed to cDNA using the iScript cDNA Synthesis Kit (Bio-Rad Laboratories, Hercules, CA, USA) in accordance with the manufacturer’s instructions. Real-time PCR analysis was performed using the SYBR Green method. The reaction setup for each diluted cDNA sample (1:10) was assembled using the iTaq Universal SYBR Green Supermix (Bio-Rad Laboratories, Hercules, CA, USA) per the manufacturer’s instructions. The oligonucleotides (MMP-12: Forward primer-5′gctagaagtaactgggcaact3′, Reverse primer-5′gagataccgcttcatccatctt3′; *18S* rRNA: Forward primer-5′acgtctgccctatcaactttc3′, Reverse primer-5′ttggatgtggtagccgtttc3′) used for the reaction were obtained from the Integrated DNA Technologies (IDT). Samples were subjected to the following PCR cycle: [95°C for 5 min, (95°C for 30 s, 59–60°C for 30 s, 72°C for 30 s) × 40 cycles, and 72°C for 5 min] in an iCycler IQ (Multi-Color Real-Time PCR Detection System; Bio-Rad Laboratories, Hercules, CA, USA). Data was collected and recorded using the iCycler IQ software (Bio-Rad Laboratories, Hercules, CA, USA) and expressed as a function of the threshold cycle (Ct), representing the number of cycles at which the fluorescent intensity of the SYBR Green dye is significantly above the background fluorescence. *18S* rRNA served as the internal standard. Relative quantification of gene expression was normalized to *18S* rRNA. The fold change in target gene expression in the test sample relative to the control sample was computed using the formula 2^ΔCt^(control)/2^ΔCt^ (test).

### Neurobehavioral evaluations

Trained researchers who were blind to treatments conducted all neurological evaluations and data collection for ischemia-induced rats. Following 2-h of transient focal ischemia, rats from experimental groups treated with M12sh or SVsh were submitted to a battery of standard neurobehavioral tests, including the modified Neurological Severity Score assessment (mNSS), adhesive tape removal test (commonly referred to as sticky-tape test), beam walk test, and accelerating rotarod test as previously described by our group (Nalamolu et al., 2021a). The mNSS assessment is a composite of reflex, balance, sensory (tactile, visual, and proprioception), and motor (abnormal movement and muscle status) functions. The sticky-tape test provides an assessment of somatosensory dysfunction. The beam walk test evaluates motor coordination and integration. The rotarod test evaluates motor coordination, balance, and grip strength. Rats were trained to traverse a narrow raised beam and walk on a rotating rotarod for 2 or 3 days before ischemia induction. By the end of the training period, all rats had learned the tasks to a satisfactory level. The tests were administered prior to ischemia (baseline) and at regular intervals (on reperfusion days 1, 3, 5, 7, 14, and 21) after ischemia to determine the time course of neurological and functional recovery following various treatments.

### Exclusion criteria

Rats that did not perform or meet the required standards on the accelerating rotarod test (the rotarod latency of minimum 50 s) after the training period and before the induction of ischemia were not included in the study. Animals that did not exhibit a neurological score of 8 or above after ischemia and reperfusion and/or a body weight loss of more than 5% relative to baseline on day 1 after reperfusion were excluded from the study. Additionally, animals with postmortem evidence of hemorrhage around the MCA were excluded from the study. Data obtained from animals that died during the study period, data from animals that did not demonstrate specific post-ischemic neurological deficits, and data identified as outliers by the Grubbs test were excluded from analysis.

### Statistics and data analysis

Statistical analysis of the data was performed using GraphPad Prism 8.4.3 for Windows. For each experiment, the quantitative data was tested for normality and equality of variances. On the basis of the number of groups present in each experiment and the outcome of the normality and variance tests, the appropriate statistical tests (described in the figure legends) were selected to analyze the data. Differences between groups were considered significant at *p* < 0.05. All data are expressed as mean ± SEM.

## Results

### Matrix metalloproteinase-12 expression increases in the brain after ischemic stroke and M12sh treatment prevents this

**FIGURE 2:**
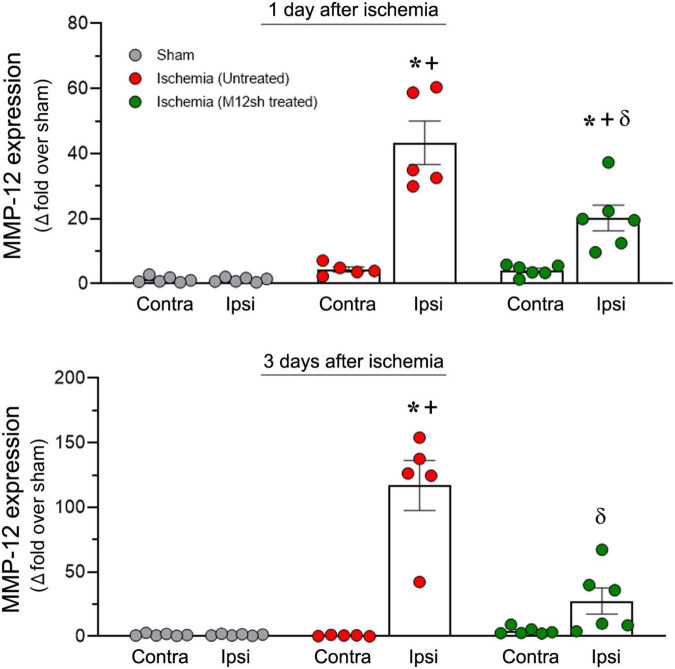
M12sh treatment is effective in reducing MMP-12 expression *in vivo*. The column scatter plots show the quantified mRNA expression of MMP-12 (expressed as fold change over sham) on post-reperfusion days 1 and 3 in the contralateral (Contra) and ipsilateral (Ipsi) brain hemispheres of male rats subjected to 2-h transient focal cerebral ischemia. Statistical analysis: Two-way ANOVA followed by Sidak’s/Tukey’s multiple comparisons tests. ^+^*p* < 0.05 vs. Contralateral brain hemisphere (same experimental group). *p < 0.05 vs. Sham (same brain hemisphere). ^δ^ *p* < 0.05 vs. Ischemia (Untreated) group (same brain hemisphere).

Real-time PCR was performed to evaluate MMP-12 mRNA expression in the ipsilateral/ischemic and contralateral brains of male rats from sham and untreated/M12sh treated ischemia-induced animals on days 1 and 3 following reperfusion. On post-reperfusion day 1, MMP-12 expression was markedly increased in the ipsilateral/ischemic brains of untreated (43-fold over sham) and to a lesser extent in M12sh-treated (20-fold over sham) ischemia-induced rats (Figure 2). Statistical analysis revealed significant effects of brain region, treatment, and brain region × treatment interaction. These results indicate that the level of MMP-12 expression varied between brain regions and was affected by treatments. A subsequent *post-hoc* test revealed that MMP-12 expression in the ipsilateral/ischemic hemisphere was significantly higher in the untreated (*p* < 0.0001) and M12sh-treated (*p* = 0.0076) groups compared to the contralateral hemisphere. In addition *post-hoc* testing revealed no significant differences between experimental groups in the expression of MMP-12 in the contralateral brain; however, in the ipsilateral/ischemic brain, MMP-12 expression was significantly increased in both the untreated (*p* < 0.0001) and M12sh-treated (*p* = 0.0002) groups compared to the sham group. These findings suggest that the effects of acute ischemia on MMP-12 expression in the brain are restricted to the ischemic hemisphere only. In addition, this test demonstrated that MMP-12 expression in the ipsilateral/ischemic brain was significantly reduced (*p* < 0.0001) by about 53% in the M12sh-treated group compared to the untreated group.

In animals euthanized three days after reperfusion, we observed a marked increase in the expression of MMP-12 in the ipsilateral/ischemic brain of untreated rats (117-fold over sham) and a relatively smaller increase in M12sh-treated (28-fold over sham) ischemic rats (Figure 2). Statistical analysis revealed a significant effect of brain region, treatment, and brain region × treatment interaction. Subsequent *post-hoc* testing revealed that MMP-12 expression in the ipsilateral/ischemic brain relative to the contralateral brain was significantly elevated only in the untreated group (*p* < 0.0001) and not in the M12sh and sham groups. In addition, *post-hoc* testing revealed no significant differences among experimental groups in the expression of MMP-12 in the contralateral brain; however, in the ipsilateral/ischemic brain, MMP-12 expression relative to the sham group was significantly elevated in the untreated group (*p* < 0.0001) but not in the M12sh group. Furthermore, this test showed that MMP-12 expression in the ipsilateral/ischemic brain was significantly reduced in the M12sh group (*p* < 0.0001) compared to the untreated group.

Overall, our results indicate that MMP-12 expression increases in the brain following an ischemic stroke, and that M12sh treatment immediately following acute ischemia is effective in preventing this increase.

### The earlier the matrix metalloproteinase-12 suppression, the better the post-stroke neurological recovery in male rats

**FIGURE 3:**
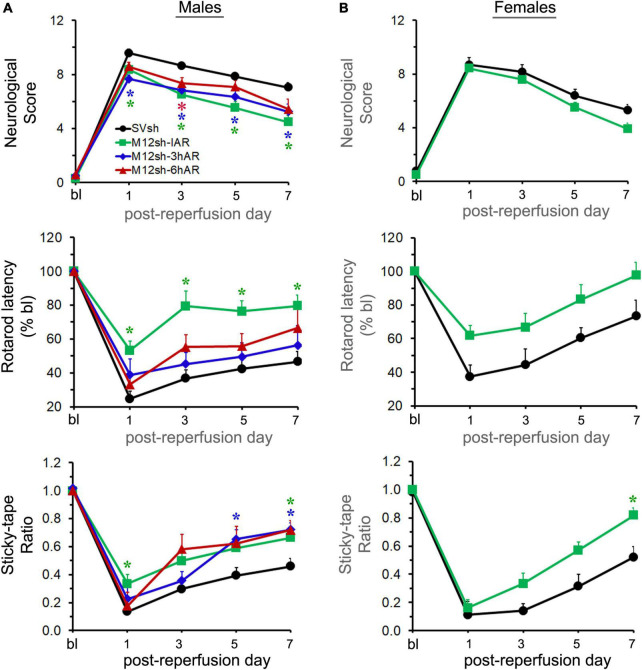
The effect of a single-dose M12sh treatment given at different times after reperfusion on post-stroke neurological and functional recovery. Following 2-h focal cerebral ischemia, both male **(A)** and female **(B)** rats subjected to treatments with either SVsh (immediately after reperfusion) or M12sh (immediately after reperfusion, 3-h after reperfusion, or 6-h after reperfusion) were evaluated for assessment of various neurological and functional tests at baseline (bl) and at regular intervals (days 1, 3, 5, and 7). Post-ischemic neurological and functional deficits were assessed by the mNSS assessment, the accelerating Rotarod performance, and the modified adhesive removal tests. *n* = 23–28 males and 11–13 females (SVsh group), 26–28 males and 20–22 females (M12sh-IAR group), 10-12 males (M12sh-3hAR group), and 12 males (M12sh-6hAR group) for the various tests. Statistical analysis: Two-way repeated-measures ANOVA followed by Dunnett’s/Sidak’s multiple comparisons tests. *p < 0.05 vs. SVsh (respective post-reperfusion day).

The mNSS score evaluates balance, sensory, and motor functions, and a number of reflexes; the greater the score the more severe the neurological deficits. As expected, the neurological scores of the male rats in all experimental groups dramatically increased after transient focal cerebral ischemia and reperfusion, and gradually declined over the course of the study in all the groups (Figure 3A). Statistical analysis revealed a significant effect of treatment. *Post–hoc* testing indicated that neurological scores were significantly lower in the M12sh-IAR group (*p* = 0.0254 on day 1, *p* = 0.0001 on day 3, *p* = 0.0002 on day 5, and *p* = 0.0003 on day 7), M12sh-3hAR group (*p* = 0.0003 on day 1, *p* = 0.0286 on day 3, *p* = 0.0488 on day 5, and *p* = 0.0398 on day 7), and M12sh-6hAR group (*p* = 0.0405 on day 3) compared to the SVsh group. In addition, the results of this test revealed that the decrease in neurological scores on post-reperfusion days 3, 5, and 7 relative to the SVsh group was statistically more significant in the M12sh-IAR group than in the other M12sh treated groups.

The rotarod test evaluates the animal’s ability to maintain movement on a rotating spindle; the shorter the latency to fall, the greater the impairment of motor function and grip strength. Following transient focal cerebral ischemia and reperfusion, the rotarod latency of male rats significantly decreased in all experimental groups, albeit to a lesser extent in the M12sh-IAR group, and gradually increased over the course of the study in all the groups, as expected (Figure 3A). Statistical analysis demonstrated a highly significant treatment effect. A subsequent *post-hoc* test revealed that rotarod latencies were significantly increased only in the M12sh-IAR group (*p* = 0.0005 on days 1, 3, and 5 and *p* = 0.0014 on day 7) compared to the SVsh group. These results indicate that the sooner M12sh is administered, the better the recovery of motor function.

The sticky-tape test evaluates somatosensory function; the lower the sticky-tape ratio (animal’s interaction time between affected and unaffected forelimbs), the worse the somatosensory function. The sticky-tape ratio of male rats dramatically decreased in all experimental groups, although to a lesser extent in M12sh-IAR, after transient focal cerebral ischemia and reperfusion, and gradually increased over time in all the groups, as expected (Figure 3A). Statistical analysis revealed a significant effect of treatment and a subsequent *post-hoc* test revealed that the sticky-tape ratio was significantly increased in the M12sh-IAR group (*p* = 0.026 on day 1 and *p* = 0.0316 on day 7), M12sh-3hAR group (*p* = 0.0211 on day 5 and *p* = 0.0038 on day 7), and M12sh-6hAR group (*p* = 0.0164 on day 7) compared to the SVsh group. Thus, all M12sh treated groups showed significantly higher sticky-tape ratios on post-reperfusion day 7 compared to the SVsh group irrespective of the timing of the treatment. Further analyses of the animal sticky-tape interaction time with the contralateral (left) forelimb revealed a significant and sustained increase only in the M12sh-IAR group compared to the SVsh group (Supplementary Figure 1). This indicates that M12sh treatment greatly improved somatosensory function in the affected forelimbs of stroke-induced rats in the M12sh-IAR group.

Overall, these results show that M12sh administration after transient focal cerebral ischemia and reperfusion in male rats promotes recovery of sensory and motor function. In addition, we found that treatment with M12sh immediately after reperfusion results in a greater recovery of sensory and motor function than delayed treatments.

### The effect of matrix metalloproteinase-12 suppression immediately after reperfusion on post-stroke neurological recovery in female rats

As we observed significant improvements in the recovery of sensory and motor function following ischemic stroke in male rats treated with M12sh immediately after reperfusion, we tested the effect of this treatment under similar experimental conditions in stroke-induced female rats to determine whether they also demonstrate significant improvements in stroke outcomes. A secondary objective was to determine if there are sex differences in treatment results. As expected, the M12sh-IAR had a higher reduction in neurological score and a greater improvement in rotarod latency and sticky-tape ratio than the SVsh group (Figure 3B). Statistical analysis revealed a significant effect of treatment only for the sticky-tape ratio. A subsequent *post-hoc* test revealed that the increase in sticky-tape ratio was statistically significant (*p* = 0.0159 on day 7) in the M12sh-IAR group compared to the SVsh group. In addition, the increase in sticky-tape interaction time of the contralateral (left) forelimb was statistically significant in the M12sh-IAR group compared to the SVsh group (Supplementary Figure 1). The absence of statistically significant changes in many of these neurological tests, despite the higher degree of recovery in M12sh-treated female rats may be related to the well-known milder impairments and faster recovery in young females compared to males after an ischemic stroke. For example, in this study, the rotarod latency of control shRNA (SVsh) treated females on post-reperfusion day 7 was improved to 73% of baseline versus 46% in males. Similarly, on post-reperfusion day 7, the rotarod latency of M12sh treated females, but not males, reached almost baseline value.

### Duration of M12sh mediated suppression of matrix metalloproteinase-12 expression in the brain after administration of a single dose of M12sh

**FIGURE 4:**
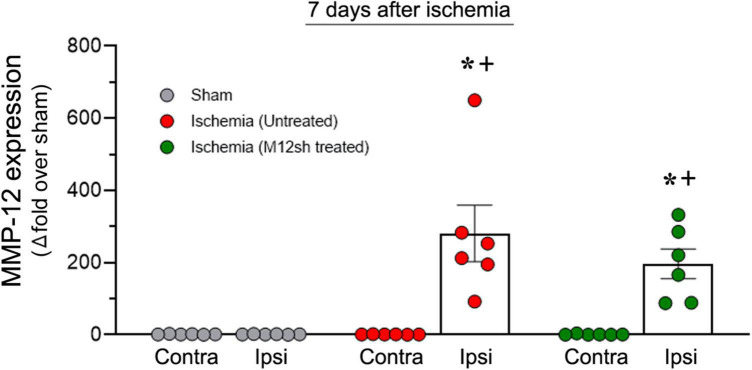
Duration of the effect of a single-dose M12sh treatment on MMP-12 expression in the brain. The column scatter plot shows the quantified mRNA expression of MMP-12 (expressed as fold change over sham) on post-reperfusion days 7 in the contralateral (Contra) and ipsilateral (Ipsi) brain hemispheres of male rats subjected to 2-h transient focal cerebral ischemia. Statistical analysis: Two-way ANOVA followed by Sidak’s/Tukey’s multiple comparisons tests. ^+^*p* < 0.05 vs. Contralateral brain hemisphere (same experimental group). *p < 0.05 vs. Sham group (same brain hemisphere).

Matrix metalloproteinase-12 mRNA expression was determined in the ipsilateral/ischemic and contralateral brain of rats from sham, and untreated/M12sh treated ischemia-induced animals euthanized 7-days after reperfusion. We observed a dramatic increase in MMP-12 expression in the ipsilateral/ischemic brains of both untreated (281-fold over sham) and M12sh-treated (197-fold over sham) ischemia-induced rats on post-reperfusion day 7 relative to days 1 and 3 (Figure 4). Statistical analysis revealed a significant effect of brain region, treatment, and brain region × treatment interaction. *Post-hoc* testing showed that MMP-12 expression in the ipsilateral/ischemic brain relative to the contralateral brain was significantly increased in both the untreated (*p* = 0.0002) and M12sh (*p* = 0.0051) groups. In addition, *post-hoc* test revealed that there were no significant differences among experimental groups in the expression of MMP-12 in the contralateral brain; however, in the ipsilateral/ischemic brain, MMP-12 expression relative to the sham group was significantly elevated in both the untreated (*p* < 0.0001) and M12sh (*p* = 0.0018) groups. Furthermore, this test indicated no significant difference in the expression of MMP-12 between the untreated and M12sh groups (*p* = 0.2442). Overall, these results indicate that M12sh treatment immediately after transient ischemia was no longer effective in reducing MMP-12 expression in the brain seven days after reperfusion.

### The effects of acute versus chronic matrix metalloproteinase-12 suppression on post-stroke neurological outcomes

While acute MMP-12 suppression was achieved by administering a single dose of M12sh immediately after reperfusion (M12sh-SD), chronic MMP-12 suppression was achieved by administering repeated doses of M12sh (immediately after reperfusion, seven days after reperfusion, and 14 days after reperfusion) (M12sh-RD). As we did not observe any statistically significant changes in neurological outcomes in the SVsh repeated-dose group relative to the SVsh single-dose group (Supplementary Figure 2), we compared the effects of M12sh-SD and M12sh-RD groups with the SVsh-SD (SVsh) group. Following transient focal cerebral ischemia, the body weight of male and female rats in all experimental groups decreased and remained below baseline levels until about day 7 post-reperfusion, and then steadily increased to levels above baseline (Supplementary Figure 3). Statistical analysis revealed no significant effect of treatment on the body weight of male or female rats any time points after reperfusion during the 21-day study period.

**FIGURE 5:**
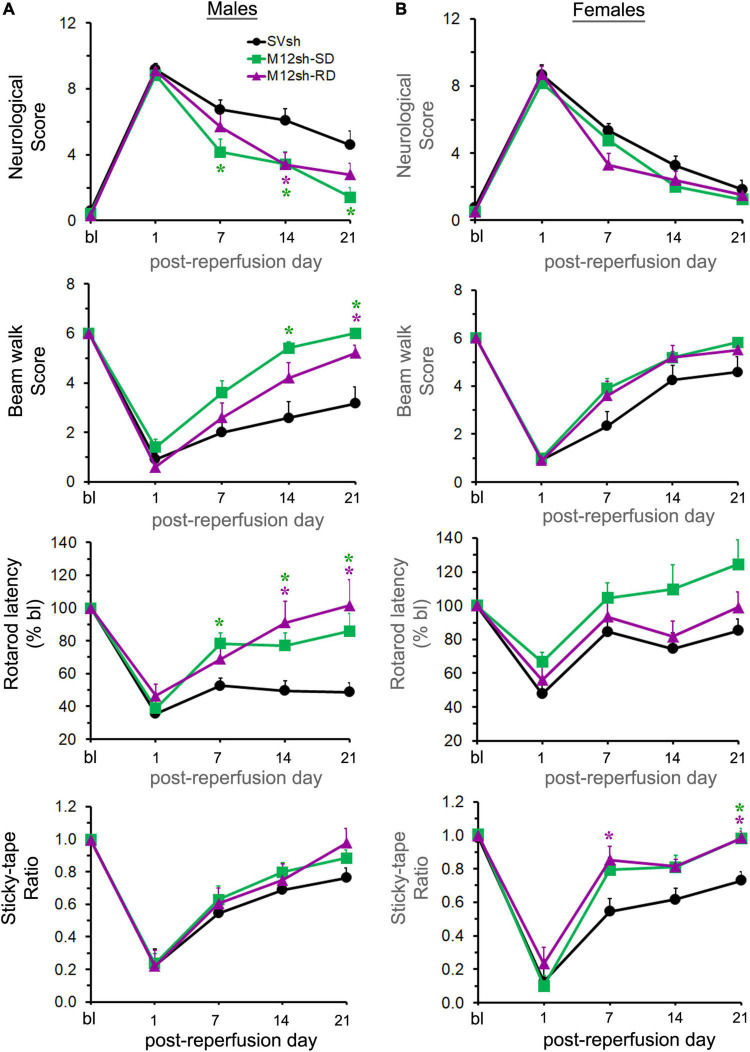
Effect of acute vs. chronic suppression of MMP-12 on long-term neurological recovery after an ischemic stroke. Following 2-h focal cerebral ischemia, male **(A)** and female **(B)** rats subjected to treatments with either single-dose (SD) SVsh/M12sh (immediately after reperfusion) or repeated-dose (RD) M12sh (immediately after reperfusion and on post-reperfusion days 7 and 14) were evaluated for assessment of neurological and functional tests at baseline (bl) and at regular intervals (days 1, 7, 14, and 21). Post-ischemic neurological and functional deficits were assessed by the mNSS assessment, the beam walk, the accelerating Rotarod performance, and the modified adhesive removal tests. *n* = 8–12 males and 10–12 females (SVsh group), 10–12 males and 9–12 females (M12sh-SD group), and 9–10 males and 10 females (M12sh-RD group) for the different tests. Statistical analysis: Two-way repeated-measures ANOVA followed by Tukey’s multiple comparisons tests. *p < 0.05 vs. SVsh (respective post-reperfusion day).

Statistical analysis of neurological scores obtained from the mNSS assessment revealed a significant effect of treatment in males but not in females (Figures 5A,B). A subsequent *post-hoc* test in males revealed that neurological scores were significantly lower in the M12sh-SD group (*p* = 0.0357 on day 7, *p* = 0.0426 on day 14, and *p* = 0.0175 on day 21) and the M12sh-RD group (*p* = 0.0384 on day 14) compared to the SVsh group. The difference in neurological scores between the M12sh-SD and M12sh-RD groups was not statistically significant at any of the time points examined.

The beam walk test was performed to determine if it can differentiate between the effects of single/repeated doses of M12sh and SVsh treatments on motor improvements. Statistical analysis revealed a significant effect of treatment in males but not in females (Figures 5A,B). *Post-hoc* testing in males indicated that beam walk scores were significantly increased in the M12sh-SD group (*p* = 0.004 on day 14 and *p* = 0.0043 on day 21) and the M12sh-RD group (*p* = 0.0415 on day 21) compared to the SVsh group. The differences in beam walk scores between M12sh-SD and M12sh-RD groups in both males and females were not statistically significant at any of the time points examined.

The rotarod latency was substantially decreased in both males and females after transient ischemia and reperfusion, and gradually increased over time (Figures 5A,B). Statistical analysis revealed a significant effect of treatment in both males and females. *Post-hoc* testing in males showed that the rotarod latency was significantly increased in the M12sh-SD group (*p* = 0.0233 on day 7, *p* = 0.0282 on day 14, and *p* = 0.0266 on day 21) and the M12sh-RD group (*p* = 0.0342 on day 14 and *p* = 0.0207 on day 21) compared to the SVsh group. Although the degree of recovery was greater in the M12sh-SD group compared to the SVsh group in females, the *post-hoc* test indicated that the treatment effects across the different experimental groups were not statistically significant. The rotarod latency was not statistically different between the M12sh-SD and M12sh-RD groups at any of the examined time points in both males and females.

As expected, the sticky-tape ratio was substantially decreased in both male and female rats after transient focal cerebral ischemia and reperfusion and gradually increased over time in all experimental groups (Figures 5A,B). Statistical analysis revealed a significant effect of treatment in females but not in males. A subsequent *post–hoc* test in females showed that the sticky-tape ratio was significantly increased in the M12sh-SD group (*p* = 0.0149 on day 21) and the M12sh-RD group (*p* = 0.0332 on day 7 and *p* = 0.0029 on day 21) compared to the SVsh group. Analysis of the sticky-tape interaction time with the contralateral (left) forelimb in males and females showed similar findings to those obtained with the sticky-tape ratio (Supplementary Figure 4). In addition, neither the sticky-tape ratio nor the interaction time with the affected forelimb differed significantly between the M12sh-SD and M12sh-RD groups at any of the time periods examined.

Overall, these results demonstrate that both the single and the repeated (once per week) doses of M12sh promote neurological recovery in both males and females after an ischemic stroke. However, the degree of improvement in females treated with M12sh relative to those treated with control shRNA was not always significant. This could be because females treated with control shRNA exhibited less neurological impairment and better recovery than males, at least in the motor tests (Supplementary Figure 5). In addition, these results indicate that continued MMP-12 suppression neither enhanced nor diminished the benefits of acute MMP-12 suppression. The improved post-stroke neurological and functional outcomes observed in this study may be attributed to the immediate MMP-12 suppression after reperfusion.

## Discussion

The Stroke Therapy Academic Industry Roundtable (STAIR) recommends that the initial evaluation of potential new stroke therapeutics be performed on young, healthy animals as was the case in our research. In this study, we showed that a gene-silencing treatment (M12sh) that blocks MMP-12 elevation in the ischemic brain promotes neurological and functional recovery in young male and female rats following an ischemic stroke. In addition, our research revealed that initiating this treatment immediately after reperfusion results in a better recovery of sensory and motor function than delayed treatments. We also found that chronic MMP-12 suppression neither enhanced nor diminished the beneficial effects of acute MMP-12 suppression, suggesting that a single-dose treatment may be sufficient for maximal therapeutic effect.

The pathologic role of MMP-12 has been demonstrated in a variety of neurologic diseases, including hemorrhagic stroke, spinal cord injury (SCI), and other pathologies (Power et al., 2003; Wells et al., 2003, 2005; Veeravalli et al., 2009; Chelluboina et al., 2018). Suppression of MMP-12 with non-specific inhibitors or genetic deletion of MMP-12 led to enhanced recovery after SCI and intracerebral hemorrhage (ICH) (Wells et al., 2003, 2005; Lee et al., 2012). Previously, our group demonstrated the deleterious role of MMP-12 in mediating BBB disruption neuroinflammation, apoptosis, and infarct development following ischemic stroke (Chelluboina et al., 2015a,b). This study highlights the enhanced neurological and functional recovery after an ischemic stroke when MMP-12 expression is suppressed.

In contrast to our previous studies, MMP-12 expression was determined in both the ipsilateral and contralateral brains of stroke-induced rats on post-reperfusion days 1, 3, and 7. As expected, MMP-12 expression was significantly increased in the ipsilateral brain but not the contralateral brain. The level of expression in the ipsilateral brain gradually increased from day 1 to day 7. These results confirm and extend our previous report on the elevated expression of MMP-12 in the ipsilateral rat brain (Chelluboina et al., 2015b). Similar time-dependent increases in MMP-12 expression were observed earlier in animal models of SCI and ICH by our group and others (Power et al., 2003; Wells et al., 2003, 2005; Lee et al., 2006; Veeravalli et al., 2009). Although various brain cells (neurons, oligodendrocytes, and microglia) express MMP-12 in the brain following an ischemic stroke, the primary source of MMP-12 is the monocytes/macrophages that have infiltrated into the ischemic brain (Wu et al., 2000; Svedin et al., 2009; Chelluboina et al., 2015b). Since the targets of M12sh treatment in this study are both the brain cells and peripheral monocytes/macrophages, M12sh was administered intravenously (rather than brain-specifically), in order to transfect both circulating blood and brain cells.

A single dose of M12sh significantly decreased brain MMP-12 mRNA expression on days 1 and 3 compared to the relative expression observed on those days in untreated rats. On day 7, the expression of MMP-12 in the ischemic brain did not differ significantly between untreated and M12sh-treated rats. These results indicated that a single-dose of M12sh administered to rats immediately after transient ischemia was no longer effective in reducing MMP-12 mRNA expression after 7 days. Our previous research showed a significant decrease in MMP-12 protein expression on day 7 following a single intravenous administration of the same dose of M12sh one day after reperfusion (Chelluboina et al., 2015b). Since we previously determined that a single dose of M12sh administered one day after reperfusion reduced MMP-12 protein expression by approximately 50% on day 7, the second and third doses of M12sh in the repeated-dose study were administered at intervals of seven days in order to achieve chronic MMP-12 suppression. Thus, stroke-induced rats in the repeated-dose group (M12sh-RD) were administered M12sh immediately after reperfusion and on days 7 and 14.

In male rats, treatment with M12sh (M12sh-IAR) led to a significant reduction in injury severity and deficits in gross motor function and sensory function during the first week following reperfusion. Furthermore, neurological scores remained significantly lower, and beam walk scores and rotarod latencies were higher in the M12sh-IAR/M12sh-SD group compared to the SVsh group on days 14 and 21 in males. In female rats treated with M12sh (M12sh-IAR), there was only a significant improvement in sensory function on days 7 and 21. Although changes in other functional tests were minimal in females, the magnitude of the reduction in neurological score, improvement in the beam walk score, and reduction in rotarod latency was greater in the M12sh-IAR group than in the SVsh group. The absence of statistical significance in the neurological and motor function outcomes between M12sh and SVsh treated females may be attributable to the intrinsically milder post-ischemic neurological deficits and better recovery in females. We recently reported that despite having similar pathological stroke lesions, the clinical manifestations of ischemic stroke were less severe in females than in males (Nalamolu et al., 2021b). We attribute this difference to the neuroprotective effects of estrogen in young females (Koellhoffer and McCullough, 2013). With a larger sample size of females, it may be possible to detect statistically significant effects in these neurological tests. Although the effects of M12sh treatment, especially on neurological and motor function recovery, were not statistically significant in females (for the reasons stated earlier), we believe that M12sh treatment is effective in both sexes. The overall findings of this study strongly support the hypothesis that suppressing MMP-12 after an ischemic stroke improves neurological and functional recovery. Using animal models of SCI and ICH, Wells et al. (2003, 2005) demonstrated a similar improvement in long-term neurological recovery in MMP-12 knockout mice compared to wild type, further supporting a predominantly pathologic role of the proteolytic enzyme in CNS injury.

Matrix metalloproteinase-12 suppression may facilitate recovery of neurological function following ischemic stroke by reducing BBB disruption, infarct volume, and brain cell death (Chelluboina et al., 2015a,b). In addition, MMP-12 suppression may improve recovery by reducing neuroinflammation after injury (Chelluboina et al., 2015b). Hohjoh et al. (2020) observed co-localization of MMP-12 with activated microglia and upregulation of MMP-12 on day 3 in a photothrombotic model of ischemic stroke. Liu et al. (2013) reported increased expression of MMP-12 in the aging brain, which correlated with decreased pro-inflammatory cytokine expression in MMP-12 knockouts. Furthermore, MMP-12 converts PGRN, a potent neurotrophic molecule produced by neurons and microglia, into granulin, a pro-inflammatory molecule (Suh et al., 2012). MMP-12 also likely contributes both directly and indirectly to demyelination, as reported by our group in the ischemic stroke model and another group in the murine encephalitis model (Noble et al., 2002; Hansmann et al., 2012).

When M12sh was administered 3 h after reperfusion, significant improvements in neurological scores were observed throughout the entire first week following reperfusion. There were also delayed improvements in sensory function on days 5 and 7. When M12sh was administered 6 h after reperfusion, the level of statistical significance decreased and largely disappeared. Compared to the control shRNA-treated rats, the changes in beam walk score and rotarod latency in animals treated with delayed M12sh were not statistically significant at any time points examined. Our investigation indicates that increasing the interval between the onset of reperfusion and M12sh delivery by three or more hours has a significant impact on M12sh’s therapeutic efficacy in males. This is probably due to ongoing BBB disruption during ischemia and the early period of reperfusion, as we have previously shown (Chelluboina et al., 2015a). As the BBB degrades, a growing number of monocytes enter the brain and differentiate into macrophages, the primary source of MMP-12 in the ischemic brain. The invading macrophages raise MMP-12 levels and exacerbate brain damage, thereby partially offsetting or obscuring the therapeutic effects of M12sh treatment. This may explain why the delayed shRNA treatments (3 and 6 h post-reperfusion in males) appear less effective and why administering shRNA treatment to females immediately following ischemia was optimal to evaluate its effects on females in this study. Overall, our results indicate that the degree of improvement depends on how quickly MMP-12 suppression is achieved after reperfusion. In addition, these findings suggest that the beneficial administration window can occur within a clinically feasible timespan after chemical thrombolysis or endovascular thrombectomy.

The long-term use of MMP inhibitors can compromise the beneficial effects of MMPs during the repair process in certain neurodegenerative diseases. For example, pharmacological blockade of MMPs by non-specific MMP inhibitors during the first three days following SCI is neuroprotective and promotes long-term recovery of hind limb function (Noble et al., 2002). However, the functional improvement was lost when MMP inhibition was continued for 7 days after SCI (Trivedi et al., 2005). The adverse effects of long-term suppression of MMPs may be attributed to the elevated expression and activity of MMP-2, which plays a role in tissue repair and nerve regeneration during the recovery or repair phase following CNS insults (Montaner et al., 2001; Goussev et al., 2003). During the acute phase of injuries such as SCI, ICH, and ischemic stroke, elevated MMP-12 is detrimental, but it aids in the recovery from experimental autoimmune encephalomyelitis (Wells et al., 2003, 2005; Weaver et al., 2005; Chelluboina et al., 2015a,b). In the present study, continued MMP-12 suppression neither enhanced nor diminished the neurologic functional benefits of acute MMP-12 suppression, suggesting that chronic suppression of MMP-12 after ischemic stroke is not detrimental to recovery. The improved neurological and functional outcomes following ischemic stroke observed in this study may be attributable to acute MMP-12 suppression. Unlike other MMPs, the benefits of MMP-12 suppression may not be temporally limited to a specific time frame and because MMP-12 may only have a pathological function during ischemic stroke and recovery (Montaner et al., 2001; Goussev et al., 2003; Lee et al., 2006; Zhao et al., 2006).

Age and sex hormones are well-known risk factors for stroke in both humans and animals. In accordance with STAIR recommendations, young male and female rats were used in this study as opposed to older animals. The therapeutic benefits of MMP-12 shRNA treatment, especially on neurological and motor function recovery, were more apparent in young males than in young females, presumably because high estrogen levels in females are associated with stroke neuroprotection. We hypothesize that the therapeutic effects of suppressing MMP-12 using shRNA will be significantly greater in stroke-induced aged animals, especially in postmenopausal females with low estrogen levels. This is because older animals, in general, are more susceptible to stroke and recover more slowly, making it easier to observe favorable treatment outcomes. Our future studies will examine the effects of suppressing MMP-12 on post-stroke brain damage and neurological recovery in aged animals as well as in animals with co-morbidities, such as hypertension and diabetes.

One of the drawbacks of our current study is a lack of neurocognitive testing to evaluate learning and complex task management, which we would like to implement in the near future. This may entail extending the experimental timeframe in order to prevent the confounding influence of neurological deficits, as almost all memory tests (such as the Morris water maze and novel object recognition/location) have a significant motor component. Due to the lack of data on MMP-12 protein expression on days 14 and 21 following reperfusion, it is unclear whether additional suppression by our vector has a substantial effect on the continued production of this protein.

In conclusion, we have demonstrated the efficacy of our plasmid vector delivery as evidenced by reduced MMP-12 expression in the ischemic brain, quantified a lasting suppression of transcript, demonstrated improved neurological, sensory, and motor functions in both male and female rats (albeit treatment effects were less apparent in females except for the sensory function), identified the optimal timing of treatment that provides the greatest therapeutic benefit, and validated the safety profile of chronic MMP-12 suppression in an animal model of ischemic stroke.

## Data availability statement

The original contributions presented in this study are included in the article/Supplementary material, further inquiries can be directed to the corresponding author.

## Ethics statement

The animal study was reviewed and approved by the Institutional Animal Care and Use Committee of the University of Illinois College of Medicine at Peoria.

## Author contributions

KV: conceptualization. KV, JK, and DP: funding acquisition and supervision. KV, SC, CF, KN, and BW: animal surgeries and care. SC, CF, KN, BW, RM, EO, and AU: methodology, investigation, and acquisition of data. KV, CF, and SC: data analysis and interpretation. KV and BW: writing-original draft of the manuscript. CF: manuscript editing. DP, JK, SC, BW, KN, RM, EO, and AU: manuscript review. All authors approved the final manuscript.
