# Supplementary material for: Therapeutic efficacy of matrix metalloproteinase-12 suppression on neurological recovery after ischemic stroke: Optimal treatment timing and duration
Source: Front Neurosci. 2022 Oct 4;16:1012812. doi: 10.3389/fnins.2022.1012812 (PMC9577328; doi:10.3389/fnins.2022.1012812)
Supplement: Supplementary file 1 [file Data_Sheet_1.pdf]

Supplementary Material

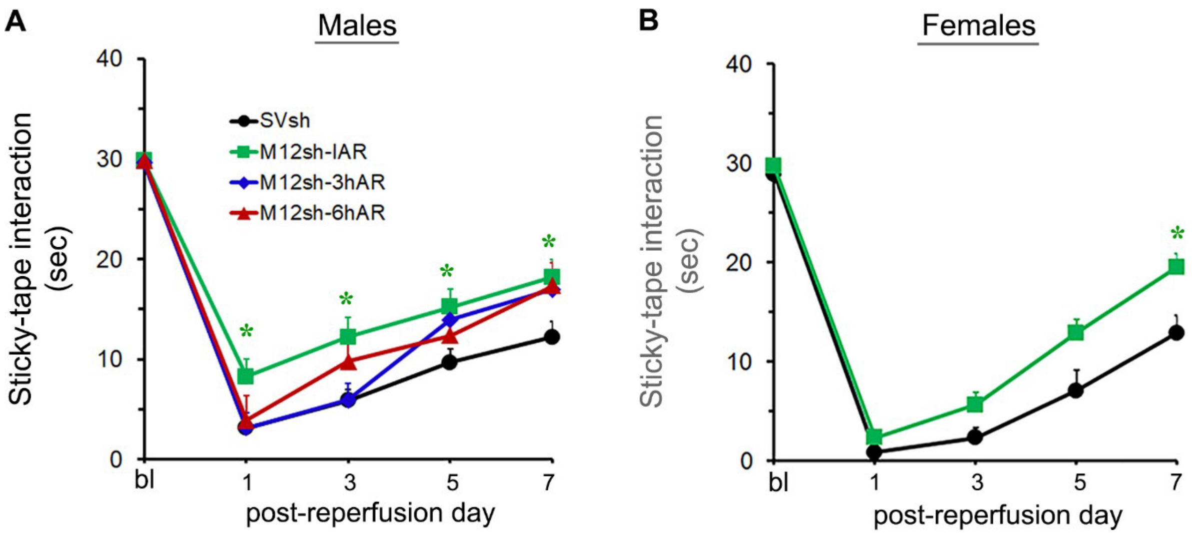

Supplementary Fig. 1. The effects of a single M12sh treatment given on different times after reperfusion on the sticky tape interaction time in male (**A**) and female (**B**) rats during the seven-day period after reperfusion. Following 2-h focal cerebral ischemia, male and female rats were subjected to treatments with either SVsh (immediately after reperfusion) or M12sh (immediately, 3-h or 6-h after reperfusion) and the sticky-tape interaction time of the left (contralateral) forelimb of the rats were evaluated at baseline (bl) and at regular intervals (days 1, 3, 5, and 7). n = 28 males and 11 females (SVsh group), 27 males and 20 females (M12sh-IAR group), 11 males (M12sh-3hAR group), and 12 males (M12sh-6hAR group). Statistical analysis: Two-way repeated-measures ANOVA followed by Dunnett's/Sidak's multiple comparisons test. *$p < 0.05$ vs. SVsh (respective post-reperfusion day).

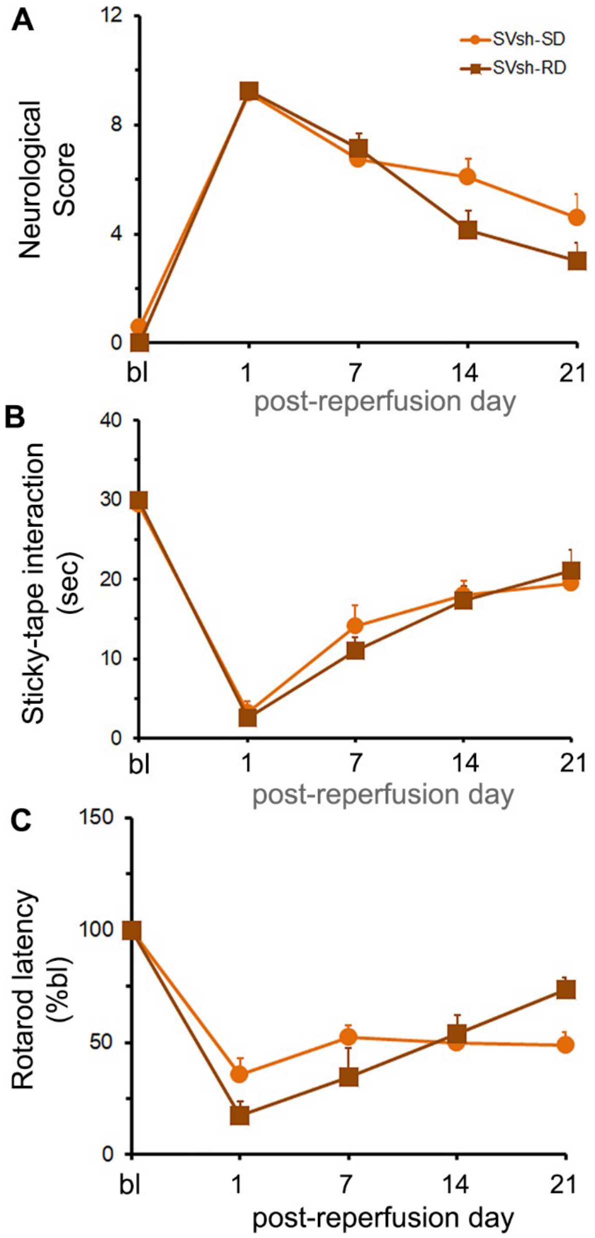

Supplementary Fig. 2. Effect of single and repeated dose SVsh treatments on post-stroke neurological/functional recovery in male rats. Following 2-h focal cerebral ischemia, male rats subjected to treatments with either single dose (SD) SVsh (immediately after reperfusion) or repeated dose (RD) SVsh (immediately after reperfusion and on days 7 and 14) were evaluated in different tests at baseline (bl) and at regular intervals (days 1, 7, 14, and 21). Post-ischemic neurological scores were determined by mNSS assessment (**A**), somatosensory function by the modified adhesive removal (**B**) test, and motor function by the accelerating Rotarod performance (**C**) test. n = 8-12 (SVsh-SD group) and 8 (SVsh-RD group). Statistical analysis: Two-way repeated-measures ANOVA followed by Sidak's multiple comparisons test. There were no statistically significant effects of the treatment.

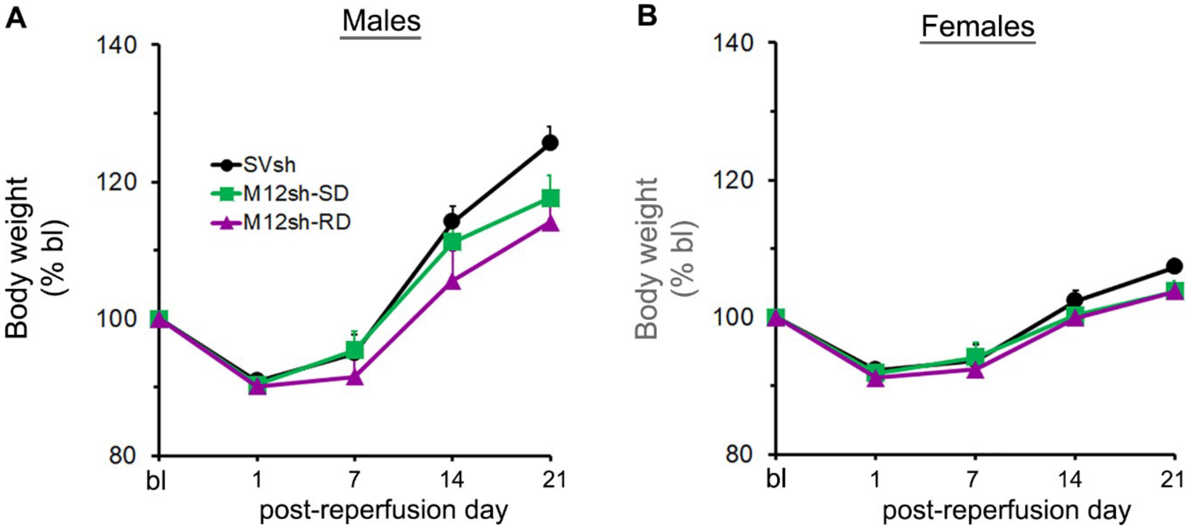

Supplementary Fig. 3. The effects of single and repeated dose M12sh treatments on post-ischemic body weight changes during the 21-day period after reperfusion. The line graphs show the changes in body weight of male (**A**) and female (**B**) rats subjected to 2-hour focal cerebral ischemia and treated with either single dose (SD) SVsh/M12sh (immediately after reperfusion) or repeated dose (RD) M12sh (immediately after reperfusion and on days 7 and 14) at baseline (bl) and at regular intervals (days 1, 7, 14, and 21). n = 12 males and 12 females (SVsh and M12sh-SD groups) and 10 males and 10 females (M12sh-RD group). Statistical analysis: Two-way repeated-measures ANOVA followed by Tukey's multiple comparisons test. There were no statistically significant effects of the treatments on body weight.

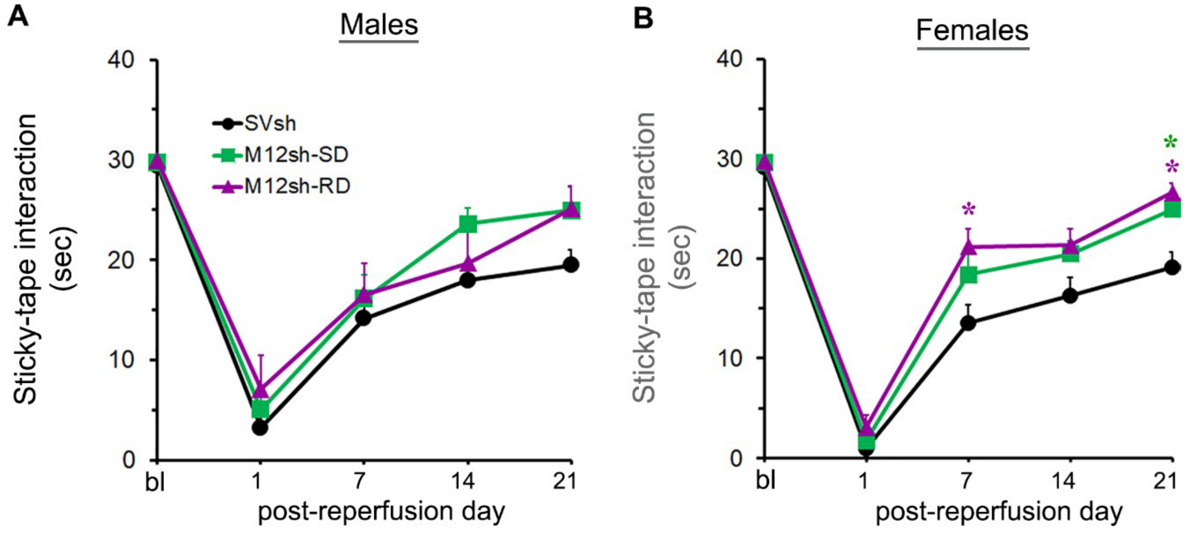

Supplementary Fig. 4. The effects of single and repeated dose M12sh treatment on the sticky tape interaction time in male and female rats. Following 2-h focal cerebral ischemia, male (**A**) and female (**B**) rats were subjected to treatments with either single dose (SD) SVsh/M12sh (immediately after reperfusion) or repeated dose (RD) M12sh (immediately after reperfusion and on days 7 and 14) the sticky tape interaction time of the left (contralateral) forelimb was evaluated at baseline (bl) and at regular intervals (days 1, 7, 14, and 21). n = 12 males and 11 females (SVsh group), 11 males and 9 females (M12sh-SD group), and 9 males and 10 females (M12sh-RD group). Statistical analysis: Two-way repeated-measures ANOVA followed by Tukey's multiple comparisons test. *$p < 0.05$ vs SVsh (respective post-reperfusion day).

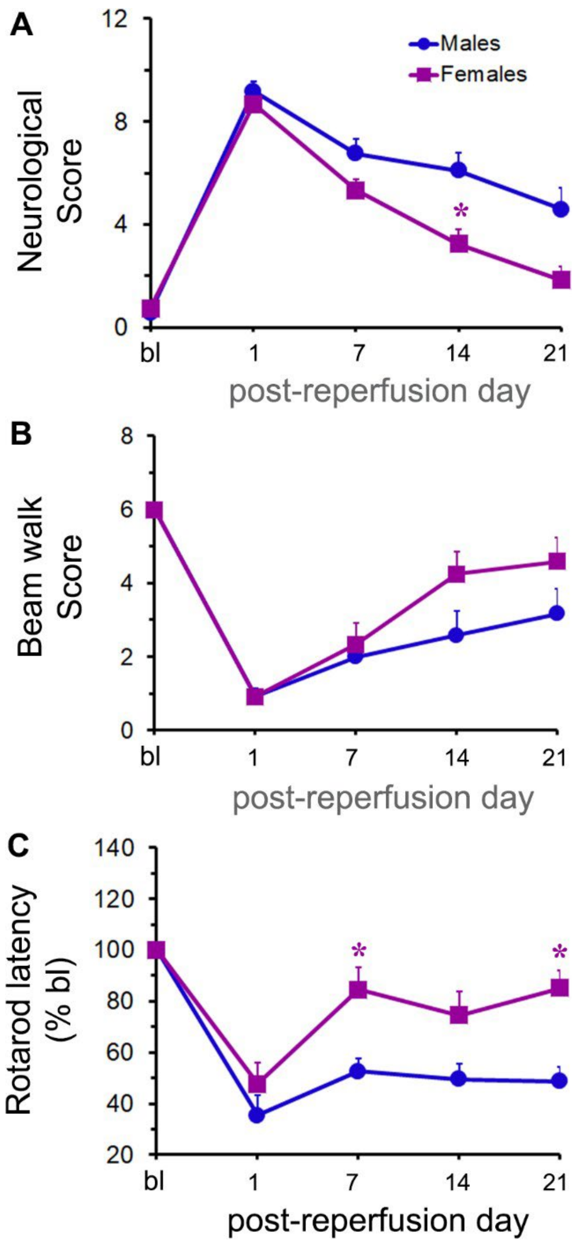

Supplementary Fig. 5. Sex differences in post-stroke neurological outcome. Following 2-h focal cerebral ischemia, male and female rats treated immediately after reperfusion with a single dose control shRNA (SVsh) were evaluated for assessment of neurological and functional tests at baseline (bl) and at regular intervals (days 1, 7, 14, and 21). Post-ischemic neurological and functional deficits were assessed by the mNSS assessment (A), the beam walk (B), and the accelerating Rotarod performance (C) tests. n = 8-12 males and 10-12 females for the different tests. Statistical analysis: Two-way repeated-measures ANOVA followed by Sidak's multiple comparisons test. *$p < 0.05$ vs males (respective post-reperfusion day).
